# Cytoskeletons in the Closet—Subversion in Alphaherpesvirus Infections

**DOI:** 10.3390/v10020079

**Published:** 2018-02-13

**Authors:** Christopher E. Denes, Monica Miranda-Saksena, Anthony L. Cunningham, Russell J. Diefenbach

**Affiliations:** 1 Centre for Virus Research, The Westmead Institute for Medical Research, The University of Sydney, Westmead, NSW 2145, Australia; cden1166@uni.sydney.edu.au (C.E.D.); monica.saksena@sydney.edu.au (M.M.-S.); tony.cunningham@sydney.edu.au (A.L.C.); 2 Department of Biomedical Sciences, Faculty of Medicine and Health Sciences, Macquarie University, Sydney, NSW 2109, Australia

**Keywords:** alphaherpesvirus, cytoskeleton, actin, microtubules, intermediate filaments, herpes simplex virus, virus transport

## Abstract

Actin filaments, microtubules and intermediate filaments form the cytoskeleton of vertebrate cells. Involved in maintaining cell integrity and structure, facilitating cargo and vesicle transport, remodelling surface structures and motility, the cytoskeleton is necessary for the successful life of a cell. Because of the broad range of functions these filaments are involved in, they are common targets for viral pathogens, including the alphaherpesviruses. Human-tropic alphaherpesviruses are prevalent pathogens carried by more than half of the world’s population; comprising herpes simplex virus (types 1 and 2) and varicella-zoster virus, these viruses are characterised by their ability to establish latency in sensory neurons. This review will discuss the known mechanisms involved in subversion of and transport via the cytoskeleton during alphaherpesvirus infections, focusing on protein-protein interactions and pathways that have recently been identified. Studies on related alphaherpesviruses whose primary host is not human, along with comparisons to more distantly related beta and gammaherpesviruses, are also presented in this review. The need to decipher as-yet-unknown mechanisms exploited by viruses to hijack cytoskeletal components—to reveal the hidden cytoskeletons in the closet—will also be addressed.

## 1. Introduction

The cytoskeleton forms an integral part of eukaryotic cell structure and comprises three main classes: actin filaments, microtubules and intermediate filaments. Actin filaments are involved in internalisation of membrane vesicles, protrusion of filo- and lammellipodia, chemotaxis, cytokinesis, muscle contraction, cell migration and regulation of cellular spatial organisation [1,2,3]. Microtubules direct intracellular transport, cell motility and during cell division form the mitotic spindle [4]. Intermediate filaments (a metazoan-specific cytoskeletal structure) provide mechanical strength and structural stress protection with a transcellular network of rigid and flexible filaments [5,6].

Due to their varied and important roles in the cell, cytoskeletal filaments and their accessory proteins are common targets for intracellular human pathogens, some of which are known to subvert the cytoskeleton to effect replication or spread between cells [7,8,9,10,11]. They do this by producing proteins that interact directly with cytoskeletal filaments or by interacting with accessory proteins which drive trafficking mechanisms or alter cytoskeletal filament dynamics. Some express proteins that interact with members of cytoskeletal-dependent molecular motor superfamilies (myosins, kinesins and dyneins) for intracellular transport. Most of these interactions support entry, egress and/or intracellular transport of partially or fully assembled pathogenic particles.

We know much about these cytoskeletal components in the context of the host cell but research into viral subversion mechanisms is still somewhat in its infancy. In the context of human-tropic viruses such as alphaherpesviruses, there has been some significant advancement, particularly in our understanding of how this class of virus hijacks host cell transport processes but less is known regarding specific interactions involved in modulating cytoskeletal filaments. This review aims to discuss the current understanding of alphaherpesvirus-host cytoskeletal interaction and regulation and identify gaps in the field that still need to be addressed.

## 2. Herpesviruses

### 2.1. Introduction

The family *Herpesviridae* [12] within the order *Herpesvirales*, includes over one hundred viruses, of which eight have known tropism for humans: herpes simplex virus type-1 (HSV-1), herpes simplex virus type-2 (HSV-2), varicella-zoster virus (VZV), Epstein-Barr virus (EBV), human cytomegalovirus (HCMV), human herpes virus 6 (HHV-6), human herpes virus 7 (HHV-7) and Kaposi’s sarcoma-associated herpesvirus (KSHV). These viruses are further divided into three subfamilies (*Alpha*, *Beta* and *Gammaherpesvirinae*) based on genomic sequence, biological characteristics and the cell types in which they can establish latency [13]. The remainder of this review will focus primarily on those members within the *Alphaherpesvirinae* (also termed alphaherpesviruses) subfamily, which include HSV-1, HSV-2 and VZV. Where relevant, reference to other herpesviruses, both human-tropic and nonhuman-tropic, within the three subfamilies is also included.

### 2.2. General Properties of Alphaherpesvirinae

Human-tropic *Alphaherpesvirinae* enter the body at the site of primary infection: typically, the oropharyngeal mucosa for HSV-1, the anogenital mucosa for HSV-2 and for VZV the mucosal epithelial cells of the respiratory tract. Next, the virus transits from the axon termini to the cell body (retrograde direction) of a connected sensory neuron to establish latency (in the dorsal root ganglia, or, for HSV-1, in the trigeminal ganglia) [13,14]. In these neurons, only a limited set of latency-associated transcripts (LATs) are expressed by HSV-1 genomes, with a broader range of transcripts present during VZV latency [15]. While the virus does not replicate, viral genomes are maintained as an episomal copy within the nucleus of non-dividing neuronal cells. With limited viral protein production, these cells are less vulnerable to immunological surveillance. Upon cellular stresses, latent virus can reactivate and trigger the replication and production of viral particles that travel in an anterograde direction down the axon to replicate in epithelial cells and be shed in oral or genital secretions which may result in clinical presentation of disease. Reactivation is triggered either by a local (e.g., tissue damage or ultraviolet radiation) or systemic stimulus (e.g., fever, emotional stress) [16]. Given the length of a sensory neuronal axon can be more than a metre, the role of active-directed long distant axonal transport of alphaherpesvirus is crucial to viral replication.

Between the two genera (*Simplexvirus* which includes HSV-1 and HSV-2 and *Varicellovirus* which includes VZV), differences exist at multiple levels [17]. At the genomic level, VZV has two origins of replication while HSV has three; multiple genes exist only in HSV while others exist only in VZV [18,19] and while many genes between the viruses are homologous in function, they cannot complement in vitro. The genera are also biologically distinct, as during in vivo infection, HSV-1 and HSV-2 tropism is restricted to epithelial cells of the oropharyngeal, respiratory and genital mucosae and latency is established in trigeminal ganglia [20]. In vitro, however, these viruses can penetrate a multitude of established immortalised cell lines. In contrast, VZV infects a smaller range of cell types in vitro but in vivo also infects cells of the immune system (monocytes, T- and B-lymphocytes) [21]. VZV spreads only cell-to-cell while HSV can also spread through the extracellular fluid. HSV-1/2 infects only the epidermis while VZV extends into the dermis. Both establish latency in dorsal root ganglia. HSV is spread by contact with vesicular fluids while VZV is transmitted by airborne oro-respiratory droplets. Orogenital shedding of HSV occurs frequently during asymptomatic reactivation but shedding of asymptomatic VZV in saliva is rare [22].

Clinically, HSV manifests in healthy individuals as ocular, orolabial or genital lesions but is implicated in neonatal herpes simplex or encephalitis in the immunonaïve or immunocompromised populations. The recurring infections are usually limited to a region of the affected skin/mucosa. VZV, the causative agent of chicken pox, impacts larger regions of the skin and during reactivation often causes zoster rash in whole dermatomes [23].

While much differs between the viruses of the *Alphaherpesvirinae* subfamily, there is enough similarity to make the study of homologous proteins a priority in further developing our understanding of trafficking and replication during the viral lifecycle. For all herpesviruses, however, replication and packaging of the genome occurs in the host cell nucleus, so transport mechanisms are necessary to traffic viral capsids containing the viral dsDNA genome (nucleocapsid) to and from the nucleus. Passive diffusion of capsids in the cytoplasm is inherently inefficient due to the crowdedness of proteins in the cytoplasm; it has been proposed that it would require more than 200 years to elapse before a HSV capsid had diffused 1 cm in the cytoplasm [24] and up to 1000× longer along the longest nerve! Therefore, the investigation of mechanisms employed by these viruses in facilitating transport is necessary to develop our understanding of their replication and so they may be targeted with novel antivirals.

For HSV, the emerging threat of antiviral resistance among viral isolates in immunocompromised patients has created a recent push for the development of new anti-herpetic compounds [25,26]. Current antivirals target DNA replication during the viral lifecycle but new mechanisms and the advent of combination therapies may lead to better patient outcomes. Furthermore, HSV has been targeted for use as a viable gene therapy vehicle and is used in the field of oncolytic virotherapy for the treatment of solid tumours [27,28]. Therefore, further investigation of the crucial virus-host interactions involved during the lifecycles of these alphaherpesviruses will pull the cytoskeletons from the closet; the discovery of these unknown mechanisms will benefit the understanding of alphaherpesvirus reactivation pathways and potentially lead to novel therapeutic options.

### 2.3. Maturation Cycle of Alphaherpesvirinae in Non-Neuronal Cells

**Figure 1:**
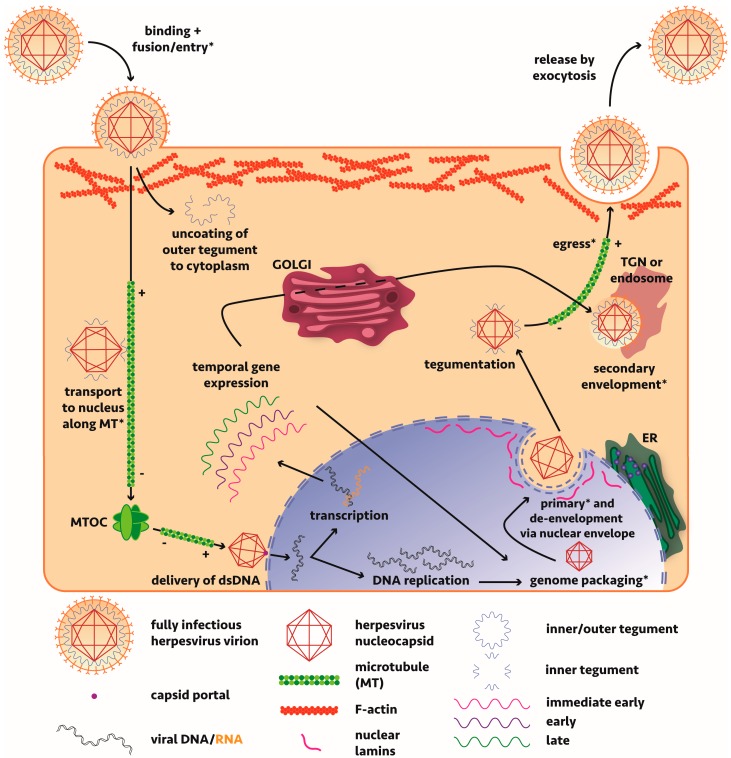
Model of alphaherpesvirus entry, assembly and egress in non-neuronal cells. Alphaherpesviruses bind host cell receptors present on the cell surface. Numerous viral glycoproteins facilitate binding and subsequent fusion of the membranes for delivery of the tegument-wrapped nucleocapsid into the cytosol. An endocytosis entry pathway exists in certain cell types (not shown here). Much of the outer tegument layer is lost to the cytoplasm but dynein motors are recruited to capsid-bound inner tegument proteins to facilitate transport in a retrograde manner along microtubules towards the microtubule organising centre (MTOC). Here, kinesin motors probably take over and transport the nucleocapsid to a nuclear pore for delivery of viral DNA to the nucleus (shown in blue) through a capsid portal. Upon entry into the nucleus, viral DNA undergoes transcription and replication. Transcript mRNA is translated in the cytosol in a temporal manner with immediate early, early and late stage proteins involved in key maturation stages. Following DNA replication, the genome is packaged inside an immature capsid before undergoing nuclear egress. The widely-supported hypothesis for nuclear egress involves budding into the perinuclear space (attaining a primary envelope) before deenvelopment by fusion with the outer nuclear membrane and release into the cytoplasm. Once here the nucleocapsid matures further and attains its tegument layer from proteins in the cytoplasm and envelope proteins processed via the endoplasmic reticulum (ER)/Golgi. Transport by kinesin motors along microtubules via the trans-Golgi network (TGN) or endosome, where the virus obtains its final host-derived envelope in a process of secondary envelopment, occurs before the virus is exocytosed for release. Stages during which remodelling and/or transport along cytoskeletal filaments may be involved are marked with an asterisk. Microtubule polarity is shown with “+” and “−” signs indicating the plus- or minus-ended nature of the microtubule tip.

A significant amount of work has been undertaken to resolve the molecular events governing alphaherpesvirus maturation and assembly with most studies based on either HSV-1 or the porcine-tropic pseudorabies virus (PrV) (Figure 1). An overview of the lifecycle of these viruses, including discussions on the mechanisms of viral maturation and egress can be found in a number of reviews [14,29,30,31,32,33,34,35,36,37]. Briefly though, the virus, consisting of a nucleocapsid surrounded by a protein tegument layer further wrapped by a host-derived envelope, enters the cell via membrane fusion mediated by viral glycoprotein binding of host surface receptors or endocytosis (cell-line dependent mechanism). The nucleocapsid, surrounded by inner tegument proteins pUS3, pUL36 and pUL37 [38,39,40], having lost most of its outer tegument to the cytosol, is transported along microtubules via tegument protein-interacting dynein motors to the nucleus [41]. The genome is passed from the viral capsid portal through a nuclear pore and subsequent replication and transcription of viral genes occurs. Capsid assembly and packaging of the genome occurs in the nucleus before the nucleocapsid undergoes nuclear egress. Two opposing mechanisms have been proposed: a primary envelopment-deenvelopment-secondary envelopment process where the nucleocapsid buds through the inner nuclear membrane or egress via a secretory pathway where it maintains its envelope. Overwhelming evidence supports the first model. Tegumentation (whereby the virus acquires all viral proteins that form the tegument matrix, primarily in the cytoplasm) occurs with viral tegument protein pUL36 pivotal in driving transport to sites of tegument addition [42,43], before the virus takes on a secondary envelope derived from the trans-Golgi network (TGN) or endosome. With much redundancy in the interactions, tegument proteins anchor nucleocapsid (inner tegument) and cytoplasmic tails of envelope glycoproteins (outer tegument) and define the maturation state of a viral particle; without the correct tegument components in vivo, the virus cannot efficiently egress. Lastly, the virus must be transported to the plasma membrane for subsequent release by exocytosis. For detailed discussion of the maturation of alphaherpesviruses in neuronal cells, the reader is referred to our companion review [44].

The viral proteins and host machinery involved are minimally understood and much remains to be discovered about virus-host interactions required during replication of alphaherpesviruses.

## 3. Filamentous Actin

### 3.1. Introduction

Filamentous actin has many roles including maintenance of cell integrity, muscle contraction and formation of stress fibres, cell protrusions (filopodia and lamellipodia) and the perinuclear and cell cortex [1]. Involved in so many processes, actin requires tight regulation by the cell. Actin exists within the cell in two forms: globular monomeric G-actin and filamentous F-actin. Filaments are polarised, with new ATP-bound actin monomers added at the “plus” or “barbed” end and ADP-bound actin lost at the “minus” or “pointed” end [45,46]. While G-actin is abundant and filaments are self-assembling, the initial nucleation step, whereby a new filament is established from G-actin monomers, is rate-limiting. Proteins and protein complexes that govern nucleation and actin dynamics are therefore critical for many cell functions.

Systems that regulate actin dynamics will be discussed briefly here but for a recent in-depth review on actin roles, assembly, regulation and nucleation mechanisms, please see [2]. There are two well-characterised regulatory mechanisms to initiate *de novo* actin nucleation: formins and the Arp2/3 complex.

Formins are dimeric membrane-bound proteins that interact with actin filaments to increase the rate of barbed end elongation while preventing the activity of capping proteins, allowing continuous extension and “pushing” of membranes for filopodia development [47]. Formins produce unbranched actin filaments and FH2 domains within formins dimerise and act as nucleators for these filaments, remaining attached for the lifespan of the filament.

The Arp2/3 complex is a heptameric protein complex, comprising actin-related proteins Arp2 and Arp3 and five additional subunits [48], which initiates the formation of branched actin networks [49] for endocytosis and motility [50]. Arp2 and Arp3 provide a nucleation template for elongation at a 70° angle from a mother filament [51,52,53,54]. This branching system has been termed the “dendritic nucleation model” of actin polymerisation and gives rise to lamellipodia [49].

Normally in an inactive state, the Arp2/3 complex requires activation from nucleation promoting factors (NPFs) such as the Wiskott-Aldrich Syndrome protein (WASP) or WASP-family verprolin-homologous protein (WAVE) families of proteins [51]. NPFs are critical for the regulation of cellular actin dynamics [55], inducing a further conformational change to the Arp2/3 complex to allow activation [51,56]. NPFs exist in the cell in an autoinhibited conformation, with this state reversed by the competitive binding of Cdc42 (a Rho-family GTPase) and phosphatidylinositol(4,5)-bisphosphate (PtdIns(4,5)*P*_2_, PIP_2_) [57].

Due to the unfavourability of spontaneous actin nucleation, pathogens that require the actin cytoskeleton for efficient replication and spread need to subvert the protein complexes involved in regulating actin dynamics [7,58]. Some pathogens produce homologues of host cell proteins involved in actin regulation. For example, *Listeria monocytogenes*, a virulent foodborne bacterial pathogen that causes listeriosis, expresses ActA, a mimic of the host NPF N-WASP, to trigger Arp2/3 complex-mediated actin regulation and facilitate pathogen movement within the cytoplasm [59]. Other pathogens are capable of triggering actin regulation by expressing proteins that can recruit NPFs. For example, vaccinia virus, the virus used as the live vaccine for smallpox, triggers Arp2/3 complex-mediated formation of actin “comets” for intracellular transport. Phosphorylation of vaccinia protein A36 recruits N-WASP and, by proxy, the rest of the complex [60]. These examples demonstrate how actin dynamics can be exploited for intracellular movement but in most cases, while many host-cell proteins have been identified as initiators of signalling pathways, not all viral binding partners have been clearly identified.

### 3.2. Actin Remodelling by Alphaherpesviruses

A number of studies have implicated actin remodelling by alphaherpesviruses during host cell entry, assembly and egress [61,62,63,64,65,66,67,68,69] (and most recently reviewed in [70]). This would be most relevant in the context of viral replication during early entry and late egress phases which require passage of virions through the F-actin cortex underlying the plasma membrane (Figure 1). Early requirements for actin remodelling are evident from the formation of filopodia expressing heparan sulphate to facilitate HSV-1 binding and subsequent lateral surfing towards the main body of the cell [71].

**Figure 2:**
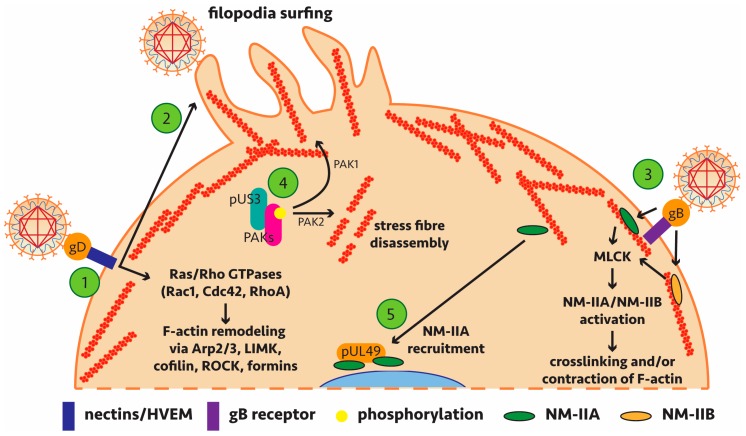
Identified alphaherpesvirus-host interactions involved in actin remodelling or myosin recruitment. This schematic highlights the findings of Table 1 and Table 2, representing the known specific interactions and pathways involved in alphaherpesviral regulation of actin dynamics. 1: Viral envelope glycoprotein gD binds nectins and/or the herpesvirus entry mediator (HVEM) to activate Ras/Rho GTPases. This triggers actin remodelling via the Arp2/3 complex, LIM domain kinases (LIMK), cofilin, Rho-associated protein kinase (ROCK) and formins. 2: gD binding to surface receptors can trigger the formation of filopodia. The virus can associate with these and undergo “surfing” to bring the virus closer to the main body of the cell for entry. 3: Non-muscle myosins, NM-IIA and NM-IIB, can act as entry coreceptors for viral envelope glycoprotein gB, activated by upstream myosin light chain kinase (MLCK) activation, leading to remodelling of F-actin. 4: Viral kinase pUS3 phosphorylation of the p21-activated kinases (PAKs) can lead to formation of protrusions (PAK1) and stress fibre disassembly (PAK2). 5: Viral protein pUL49 can sequester a subpopulation of NM-IIA at the perinuclear space which may rearrange actin for viral nuclear egress.

Of particular note, recent findings have shown dynamin dependence for HSV-1 transport to the nucleus during entry and for virus protein transport from the nucleus to cytosol following genome packaging [72]. Dynamin is a GTPase with functions in actin assembly and reorganisation and while no interaction partner was confirmed, it appears this recruitment is important for HSV-1 cell-to-cell spread. Pentagalloylglucose (PGG) treatment or targeted knockdown to down-regulate the expression of the actin depolymerising factor cofilin-1 inhibited entry of HSV-1. In this case, viral regulation of actin remodelling most likely proceeds via signalling through Ras/Rho GTPases (Rac1 and Cdc42) [73,74]. Further studies have identified (or implicated) specific viral proteins which influence actin regulatory mechanisms—leading to subversion of host machinery and signalling pathways—and play a role in alphaherpesviral replication (summarised in Table 1 with key interactions highlighted in Figure 2).

Of special interest is the viral kinase pUS3 which to date is the only alphaherpesviral protein shown to directly interact with actin regulatory mechanisms (Table 1). Commonly studied in PrV, pUS3 is conserved among the alphaherpesviruses [87] and clear binding partners involved in actin remodelling have been identified. PrV infection leads to actin stress fibre breakdown and formation of actin protrusions, all dependent on pUS3 activity and associated with increased cell-to-cell spread [81]. Comparable pUS3-induced actin network changes have been seen in HSV-2 and other alphaherpesviruses, including Marek’s disease virus (MDV), which infects chickens [84,88,89], bovine herpesvirus type 5 (BHV-5) [90] and equine herpesvirus type 1 (EHV-1) where pUS3 is also important for efficient nuclear egress [91].

More recent findings of pUS3 have shown that the p21-activated kinase PAK1 has a significant, albeit limited, role in the anti-apoptotic activity of pUS3 [92] and that pUS3 is necessary for viral passage of the basement membrane (the barrier between the epithelium and lamina propria), an important step in the early stages of herpesvirus pathogenesis [93]. As a kinase, pUS3 has been shown to phosphorylate PAK1, PAK2 and RhoA to remodel the actin cytoskeleton (Figure 2). A role for Rho/Rac signalling in remodelling of actin to facilitate spread of MDV has also been reported though a direct role for pUS3 was not determined in this case [94]. Further work is necessary to determine if these specific findings in PrV are transferable to HSV-1, HSV-2 and VZV homologs of pUS3.

### 3.3. Alphaherpesvirus Exploitation of Actin-Based Transport and Myosin

The superfamily of actin-dependent myosin molecular motors, which consists of 12 classes in humans, have a semi-conserved catalytic N-terminal head domain that possesses both actin- and ATP-binding sites [95]. Apart from muscle contraction, non-muscle myosin functions in contractile actin stress fibres, in the contractile ring during the process of cytokinesis and in the transport of cargo along F-actin. Myosins are either monomeric or dimeric in nature and travel along F-actin in an ATPase cycle-dependent manner, carrying cargo at the more variable C-terminal end [95,96]. Myosins move at varying speeds towards the plus end of F-actin with the exception being myosin VI which moves towards the minus end of F-actin. Among the myosin classes discovered, the tail regions are highly divergent [95,97] and some myosins have domains thought to facilitate particular cellular processes via adaptor or other binding proteins.

Specific myosin transport of viruses along F-actin or myosin-driven actin rearrangements to facilitate viral entry/egress would require the involvement of primarily non-muscle myosins. Activation of non-muscle myosin typically requires a phosphorylation event catalysed by enzymes such as myosin light chain kinase (MLCK) [95]. As such non-muscle myosins and their mechanism of regulation are of specific interest in the context of alphaherpesviruses. A summary of known alphaherpesvirus-myosin interactions is provided in Table 2 and key interactions highlighted in Figure 2. To date no direct interaction between an alphaherpesviral protein and a myosin motor which drives virus transport has been identified.

### 3.4. Actin Remodelling and Myosin Motor Exploitation by other Herpesviruses

Insights into the mechanisms employed by herpesviruses to regulate actin remodelling and exploit myosin can also be gained from studies on *Betaherpesvirinae* and *Gammaherpesvirinae* subfamily members. Inhibition of regulators of actin nucleation such as Rho GTPases and the Arp2/3 complex, resulting in disruption of the actin cytoskeleton, has been shown to block KSHV entry and trafficking [103]. Whether this is related to what is observed with alphaherpesviruses (Table 1) is yet to be elucidated. In the case of HCMV driven immune evasion, the viral protein pUL135 has been shown to directly interact with the WAVE regulatory complex leading to inhibition of Arp2/3 remodelling of actin and a reduced capability to form immunological synapses at the plasma membrane [104]. This is unlikely to be relevant for non-lymphotropic herpesviruses such as HSV-1 or PrV, especially considering that pUL135 is not conserved among alphaherpesviruses.

HCMV has been shown to induce nuclear actin filaments during infection to facilitate capsid nuclear egress [105]. The exact molecular mechanism is unknown but it is proposed that the virus may undergo directed transport in an F-actin dependent manner or the F-actin remodels the nuclear structure to permit efficient viral diffusion. In the same study, HSV-1 was shown not to induce these filaments in the nucleus of infected human foreskin fibroblasts, which contradicts an early study undertaken in neurons with both HSV-1 and PrV [65] which proposed Myosin Va recruitment as a mechanism of nuclear capsid motility. However, this latter group have subsequently ruled out F-actin dependent motility of nuclear alphaherpesvirus capsids [106,107], finding no F-actin in the nucleus with live cell microscopy utilising LifeAct-GFP and recognising that formation of actin rods following Latrunculin A (LatA) treatment of infected primary fibroblasts inhibits capsid motility by binding capsids to the rods [107]. This finding replaced their earlier proposal that LatA caused F-actin depolymerisation which stalled nuclear capsid motility [65] and has reopened the question into how herpesviral capsids move in the nucleus.

Myosin has also been implicated in the efficient replication of HCMV by being recruited to viral assembly sites [108]. Similar processes are likely with alphaherpesviruses but myosin class may differ according to cell type infected. A role for myosin during entry and egress of EHV-1 in primary neurons, based on myosin inhibitor studies, has been reported [109]. Furthermore, the viral thymidine kinase from KSHV has been shown to activate myosin II through RhoA-ROCK signalling leading to changes in cell morphology [110]. This process is apparently not conserved in HSV-1 [110].

### 3.5. Future Work for Alphaherpesvirus-Actin Interaction Studies

Based on the evidence presented (Table 1 and Table 2), it appears that alphaherpesviruses require actin reorganisation and exploit myosin during many phases of replication including entry and egress in the host cell cytoplasm but not during nuclear egress. Interestingly, actin can be found in purified herpesviruses, maybe also playing a structural role for the virus [64,111]. No direct viral role in regulating the pool of G-actin, which is an important factor in actin dynamics, has been reported to date. While many mechanisms of signalling and roles for host cell proteins during virus replication have been identified, it is unclear in most cases which key viral proteins are involved but indirect signals or molecular motor recruitment seem to be more common than direct subversion of the actin regulation machinery. Future work needs to be performed to identify alphaherpesviral binding partners of the myosin motors and cofilin-1 as well as to determine if human-tropic alphaherpesviral pUS3 demonstrates homologous functions to PrV pUS3. Much remains to be uncovered in the study of herpesvirus subversion of the actin network.

## 4. Microtubules

### 4.1. Introduction

Microtubules, the filaments involved in directing intracellular transport, spatial organisation of membrane-enclosed organelles and crucial to mitotic spindle formation during cell division, are the polymerised form of tubulin, itself a heterodimer of globular proteins α-tubulin and β-tubulin [4]. Microtubules are distinctly polar, with new dimers added/lost rapidly at the dynamic “plus” end (head) while the “minus” end (tail) remains stable and tethered to a microtubule organising centre (MTOC) situated at the centrosome (Figure 1). Microtubules are hollowed structures made of thirteen protofilaments to give an outer diameter of 25 nm [112]. Each protofilament is formed by the head-to-tail association of αβ dimers, with α-tubulins exposed at the tail end and β-tubulins at the head. Mammalian cells possess at least six isoforms of α- and β-tubulins, encoded by multiple genes.

While F-actin dynamics are driven by ATP/ADP, microtubule dynamics are driven by GTP/GDP. GTP can associate with β-tubulin at the plus end and if addition of subunits is rapid, hydrolysis to GDP cannot occur and the head of the microtubule remains in the “T form” (β-tubulin in association with GTP). If, however, subunit addition slows down, hydrolysis of GTP can occur and the head of the microtubule converts to the “D form” (β-tubulin in association with GDP). GTP-bound tubulin will continue to polymerise and GDP-bound tubulin will depolymerise; thus, hydrolysis leads to shrinkage of the microtubule. Even with a constant concentration of free subunits within the cytosol, the conversion between T and D forms (growing and shrinking) can happen endlessly and this process is called dynamic instability.

There are several classes of microtubule-binding proteins which also modulate filament dynamics and organisation (reviewed in [4,112]). In a similar fashion to F-actin nucleation, nucleation of microtubules is energetically unfavourable in vivo and requires an initiation mechanism. Nucleation occurs from the minus end and is dependent on γ-tubulin ring complexes (γ-TuRCs, comprising γ-tubulin, γ-tubulin complex proteins and neural precursor cell expressed developmentally down-regulated gene-1 (NEDD1)) whose 13-fold symmetry acts as a scaffold for α/β tubulin dimers to bind [113]. The γ-TuRC remains bound to the minus end of the microtubule in the centrosome of animal cells, recruited by NEDD1 [114] and acts as a cap to provide filament stability. For other recent reviews on microtubule dynamics, see [115] and [4] and for exploitation by viruses, see [116].

### 4.2. Microtubule Remodelling by Alphaherpesviruses

During viral infection, it is well documented that alphaherpesviruses remodel the microtubule network of the host cell, often observed as a loss and reformation of the MTOC [61,67,117,118,119,120]. While the human neurotropic alphaherpesviruses do infect similar cells during their lifecycles, to date few viral proteins having roles in observed microtubule remodelling have been identified and rather more viral proteins likely to function in engaging microtubule-dependent molecular motors during viral transport have been elucidated (see Section 4.3). Some interactions which stabilise or destabilise microtubules, to enhance microtubule-dependent viral transport, viral entry and egress have been identified (Table 3).

While a direct interaction has not been discovered, recent findings by Pasdeloup et al. show that HSV-1 infection triggers remodelling of the microtubule network so that the centrosome is no longer the primary MTOC during egress in the proposed pathway of nucleus to centrosome to site of secondary envelopment [117]. While the centrosome remains the MTOC in PrV-infected cells, microtubules that form late in HSV-1 infection arise from dispersed localisations within the cytoplasm. This suggests the presence of minor alternative MTOCs and the authors propose this reorganisation facilitates direct capsid transport to sites of secondary envelopment after nuclear egress.

### 4.3. Alphaherpesvirus Exploitation of Microtubule-Based Transport Motors

Due to their polar nature, microtubules are well-suited as the highways of the cell, allowing two-way traffic. The requirement for intact microtubules in the context of alphaherpesvirus infection of the host has been well established [126,127,128] (Figure 1). Motor proteins that associate with microtubules belong to two major superfamilies: the kinesins and the dyneins. Kinesin superfamily proteins (or KIFs) mediate plus-end directed motility (with some capable of minus-ended motility) [129,130] and dynein family proteins mediate minus-ended motility [131,132]. Pathogens commonly require kinesin/dynein intracellular transport and as such, mechanisms of subversion of these molecular motors have been adopted by many viruses including alphaherpesviruses [14,24,33,35,133,134,135,136].

The kinesin superfamily contains 15 smaller families, based on phylogenetic differences [137]. Among these, three kinesin types emerge based on the location of the conserved motor domain which contains the microtubule-binding site and the ATPase catalytic domain: N-kinesins with a motor domain at the N-terminus which drive plus-end directed transport (anterograde transport); C-kinesins with the motor domain located at the C-terminus which drive minus-end directed transport (retrograde transport); and M-kinesins with their motor domain in the middle of the protein which act to depolymerise microtubules [129,130]. The remaining portions of kinesin contain heptad repeats for dimerisation and more variable regions responsible for accessory light chain and/or cargo binding [129,130]. Kinesins can transport proteins or whole organelles (e.g., lysosomes, endosomes) as cargo and play important roles in cargo transport between the Golgi apparatus and the endoplasmic reticulum, as well as between the TGN and the plasma membrane and from the neuron cell body to the distal axon tip. Some cargoes contain specific binding domains for kinesins or utilise adaptor proteins for their recruitment and subsequent transport [129,130].

Dyneins are the microtubule motors responsible for retrograde transport of cargo to the minus-end of microtubules, frequently anchored at the MTOC. Dyneins form two classes based on functional/structural rules: axonemal and cytoplasmic dyneins [131]. Axonemal dyneins are involved in ciliary/flagellar beating and are not relevant to this review. Cytoplasmic dyneins, however, are responsible for intracellular transport and have roles in cell polarisation. Cytoplasmic dyneins are multisubunit proteins with two heavy chains that each contain an N-terminal base domain (to which accessory subunits bind and in turn direct binding of cargo), a motor domain and a microtubule-binding domain. The motor domains of these two-headed motors contain six AAA+ ATPase units which regulate ATP binding, hydrolysis and subsequent release of ADP and inorganic phosphate to initiate motor movement [138,139]. There are three ubiquitous regulators for dynein activity: the dynactin complex, lissencephaly 1 (LIS1) and nuclear distribution E (NUDE). Like kinesins, dyneins can transport many vesicles (endosomes, lysosomes, phagosomes, etc.) and have been shown to be subverted by multiple viruses including HSV-1, HIV and vaccinia virus [135].

Two models of axonal transport of alphaherpesviruses during egress from neurons have been proposed: subassembly (naked nucleocapsid lacking envelope) vs married (enveloped nucleocapsid) [14]. With the assumption that assembly and egress directly from the cell body of neurons is the same as for non-neuronal cells (Figure 1), these two models reflect differences in the form of viral particle transported from the cell body to the axon terminus (anterograde transport). The model of transport does impact what virion components are accessible for interaction with microtubule-based motors during anterograde transport from neurons. The reader should refer to our companion review on transport of HSV-1 in neurons [44] for further discussion on this issue and the current state of play with respect to these two models.

While many interactions have been identified between alphaherpesviral proteins and host microtubule-based motor complexes in vitro, not all have been shown to be involved in transport in vivo. Often a specific in vivo role for an identified interaction has been difficult to establish simply due to likely redundancy in virus-motor engagement [14]. Known alphaherpesvirus interactions with kinesin/dynein complexes based primarily on studies with HSV-1 and PrV are listed in Table 4.

Enough evidence is now available to identify the inner conserved tegument proteins pUL36 and pUL37 as clear effectors of kinesin/dynein recruitment [42,143,144,145,155,156,157,158]. These proteins are closely associated with the capsid and accessible to motors during entry and egress of capsids lacking envelope. Key evidence from interaction studies to date (Table 4) include one recent study showing a role for the N-terminus of pUL37 in regulating retrograde transport of PrV and HSV-1 [157] and another study confirming a proline-rich region in PrV pUL36 interacts with dynein/dynactin to drive retrograde transport [144]. The viral envelope protein pUS9 has been identified as playing a role in anterograde axonal transport in both HSV-1 and PrV, through differing interactions with either kinesin-1 or kinesin-3 respectively [147,149] (Table 4).

Interestingly, it has been suggested that acidic tryptophan domains in HSV-1 pUL36 bear resemblance to tryptophan motifs that mediate binding of cargo to kinesin-1 light chain C-terminal tetratricopeptide repeats [43]. These repeats have been shown to recruit kinesin to host cell cargo [159] or vaccinia virus particles via viral protein A36 during vaccinia virus infection [160] and could have a similar role in HSV-1 infection. HSV-1 with mutant tryptophan motifs in pUL36 accumulated at the MTOC during assembly and though not yet shown this fits with being unable to recruit kinesin-1 [42,43].

### 4.4. Microtubule Remodelling by Other Herpesviruses

Few examples currently exist in the literature on remodelling of microtubules by other herpesviruses which are relevant to alphaherpesviruses. The conserved viral kinase BGLF4 from EBV, whose equivalent is pUL13 in HSV-1, was shown to phosphorylate and down-regulate the activity of stathmin [161]. The action of stathmin is to bind tubulin heterodimers and prevent their addition to growing microtubules leading to shrinkage of microtubules [162]. One example of herpesvirus-induced host cell signalling pathways in the modulation of microtubule dynamics exists for KSHV which induces Rho GTPases resulting in downstream cytoskeletal changes which enhance trafficking of viral nucleocapsids to the nucleus [163].

### 4.5. Future Work for Alphaherpesvirus-Microtubule Interaction Studies

Studies have shown that VZV infection causes the remodelling of both actin filaments and microtubules but the mechanism by which this occurs remains to be elucidated [67]. Most transport research is conducted in HSV-1 or PrV with less focus on HSV-2 and VZV. With such homology between the family members, many discoveries in these viruses may ultimately translate to the transport involved in the HSV-2 and VZV lifecycles.

Ongoing work is required to map specific domains required for microtubule or microtubule-motor interactions. Due to the conserved nature of pUL36 across the alphaherpesviruses [36], the findings of Zaichick et al. [144] suggest a similar role may be seen for HSV-1, HSV-2 and/or VZV.

Clearly, tegument proteins are important for recruitment during assembly and secondary envelopment. Alphaherpesviral glycoproteins have also been implicated in regulating binding to kinesins in neuronal cells to facilitate transport [164,165]. Further elucidation of key domains within viral interactors will usher in new drug target possibilities and better improve our understanding of the biology of these viruses.

## 5. Intermediate Filaments

### 5.1. Introduction

Present in only a subset of metazoans, intermediate filaments form the third major cytoskeletal network, named after their intermediate size among the other filaments: 10nm in diameter compared to the 6nm diameter of F-actin and 25 nm of microtubules. These provide structural support and mechanical stability for the cell. Where actin and tubulin isoforms are encoded by few genes, the various intermediate filament family proteins are derived from over 70 different genes in humans, each with their own cell type-specific functions [166]. Well known intermediate filament family members include keratin, vimentin, neurofilament, lamin A/C and desmin [167]. All members of the intermediate filament family possess conserved central α-helical domains that consist of ~40 heptad repeat motifs that facilitate coiled-coil binding of another monomer, while N- and C-terminal regions vary widely [112]. In forming filaments, a dimer pairs with another dimer in an antiparallel manner to produce a staggered apolar tetramer: the antiparallel structure produces filament ends with the same identity. Next, tetramers join together laterally; filaments comprise eight protofilaments each made of a tetramer. For reviews on intermediate filament dynamics see [5,168,169].

Much less is known about human cell intermediate filament dynamics than the actin and microtubule networks. Many functions of the F-actin and microtubule networks have no similarities in the intermediate filament system: due to their apolarity, no molecular motors exist for transport via intermediate filaments; intermediate filament assembly is spontaneous and does not rely on nucleotide availability, having no binding site for ATP or GTP [5]; intermediate filaments are usually found in the filamentous form and not in a high concentration of monomers; intermediate filament assembly is primarily regulated via phosphorylation [170]. Unlike microtubule and actin filaments, intermediate filament assembly cannot be blocked by currently available pharmaceuticals.

### 5.2. Intermediate Filament Remodelling by Alphaherpesviruses

With such a variety in the intermediate filament protein subunit family and with expression and roles as filaments dependent on cell-type, mechanisms of subversion by alphaherpesviruses will most likely have evolved to target the intermediate filament proteins expressed in neuronal and epithelial/keratinocyte cell types. To be pan-specific they may target the conserved central domain involved in the coiled-coil formation, or be involved indirectly in intermediate filament regulation by subverting kinesin/dynein transport of intermediate filament precursors along microtubules [20].

Herpesviruses in general have such broad tissue tropism that they must encounter most intermediate filament types during infection but current knowledge is limited [20]. Keratin expressed on the surface of the epithelium appears to hinder rather than promote viral entry and there is no evidence supporting a role for vimentin. Following viral entry by fusion, vimentin has been shown to be required for HCMV infection [171] but a role for alphaherpesviruses at this stage of the viral lifecycle has not been shown. Some studies have shown HSV does appear to remodel cytoplasmic intermediate filaments [172,173].

During nuclear egress (Figure 1), herpesviruses must cross the dense meshwork of the inner nuclear membrane, composed primarily of lamin A/C and lamin B and HSV-1 infection has been shown to cause remodelling of the nuclear lamina [174]. One study has shown that the mouse cytomegalovirus (MCMV, a betaherpesvirus) overcomes the intermediate filament meshwork at the inner nuclear membrane with its M50/p35 and M53/p38 proteins, recruiting cellular protein kinase C (PKC) for phosphorylation of lamins and subsequent destabilisation of the layer [175]. Analysis of the interaction between M50 and M53 has shown similar binding of UL50 and UL53 in HCMV [176] but this requires viral protein UL97 rather than PKC [177]. The homologs of M50 and M53 in HSV-1 and PrV include pUL34 and pUL31 and extensive work has shown these proteins are essential for nuclear capsid egress (reviewed in [178]). Recently, a group has shown that the γ_1_34.5 gene product of HSV-1 bridges pUL34/pUL31, cellular p32 and PKC; a virus deleted for γ_1_34.5 or missing its amino terminus is crippled [179,180]. HSV-1 pUS3 and HSV-2 pUL13 can also phosphorylate lamin A/C [181,182] but this step may be more relevant for de-envelopment rather than primary envelopment as well as delocalise emerin (key for nuclear integrity and can bind F-actin and lamins) [183]. These interactions are necessary to facilitate viral spread and efficient replication. Other work has identified roles for vimentin in the entry of HCMV by macropinocytosis [171] but evidence for a role of vimentin in alphaherpesvirus infection is still lacking.

Gene expression studies have been used to assess the effect of infection on intermediate filament synthesis. During VZV infection of human skin cells, there is down-regulation of keratin 1, -5, -6A, -17, -71 and neurofilament 3 and up-regulation of keratin 19, desmin and vimentin [184]. HSV-1 infection of human fibroblasts causes up-regulation of keratin 8, -18, peripherin and down-regulation of vimentin [185,186]. The relevance of these findings is not clear but viral infection is clearly having an indirect effect on intermediate filament expression, even if not clearly remodelling the filaments themselves.

### 5.3. Future Work for Alphaherpesvirus-Intermediate Filament Interaction Studies

Incompletely understood stages of the herpesviral lifecycle (e.g., the translocation of the genome from the capsid into the nucleus) have the potential to involve intermediate filaments. Remodelling of these filaments may play roles at all stages of the viral lifecycle but initial evidence clearly supports nuclear functions during infection [20].

## 6. Conclusions

In summary, discovery and analysis of the multivariate mechanisms of alphaherpesvirus subversion of the host cytoskeleton will improve the understanding of the lifecycles of these viruses and may well lead to the development of new treatments for herpesvirus infections. The mechanisms involved in alphaherpesvirus regulation of cytoskeletal networks can be understood through inhibitor-based, siRNA and recombinant virus studies. The use of actin or microtubule disrupting drugs which target polymerisation/depolymerisation of these filaments has facilitated the study of virus replication but as these proteins play a role in so many cellular processes this can have off-target effects leading to confusing results. This can be further confounded by the use of different cell types such as non-neuronal vs neuronal or polarised vs non-polarised which have differing arrangements of their cytoskeletal elements along with expression of different classes of molecular motors. Therefore, in the case of neurotropic alphaherpesviruses there is a need to ensure studies are conducted in a range of cell types, neuronal and non-neuronal, which are biologically relevant to establish a true picture on the role of the cytoskeleton and associated accessory proteins and molecular motors. The use of more specific inhibitors which target individual cytoskeletal accessory proteins and molecular motors would help to overcome issues with off-target effects. Clues on targeted inhibitors come from studies on treatments for cancer and neurodegenerative diseases but have the caveat that they are designed, at least for cancer, to reduce cell survival [187,188,189]. Other nuanced approaches could involve siRNA knockdown, CRISPR/Cas9 gene editing or overexpression of dominant/negative fragments to inhibit the activity of individual proteins or protein complexes within the cytoskeletal network. Mutant alphaherpesviruses, engineered with more subtle changes in individual viral proteins rather than whole viral gene deletions [157], can be generated to evaluate the role of specific interactions with the cytoskeletal network from the viral perspective.

First proposed in 1977 as an antiherpetic compound [190], acyclovir–the current first-line nucleoside analogue-based antiviral for human alphaherpesvirus infection–has low toxicity and is highly specific for viral replication. All other clinically approved antiherpetics are also nucleoside analogues that act in a similar way: valacyclovir, ganciclovir, penciclovir and famciclovir [25]. In immunocompromised patients, the prolonged exposure to these compounds can induce antiviral resistance within the individual’s virus strain and reduce the efficiency of these drugs. Therefore, there is an urgent need for the development of new antivirals for control of these potentially life-threatening infections (see review [25]) and all stages of the viral lifecycle should be targeted for the discovery of virus-host interactions that are required for replication or trafficking.

Disrupting the cytoskeleton or targeting cellular proteins which regulate the cytoskeleton as an antiviral strategy is not ideal as this would most likely affect cell host survival. Therefore, it is imperative that we enhance our knowledge on how viral proteins interact with the cytoskeleton so as to allow design of new antiviral inhibitors which target these viral proteins, thereby minimising off-target effects on the host cell. To overcome viral resistance with this strategy, multiple stages of the viral lifecycle, whether dependent on the cytoskeleton or not, would need to be targeted in a similar way to combination therapy successfully used for HIV and hepatitis C virus. The recent example of a role for an N-terminal region of PrV and HSV-1 pUL37 in regulating retrograde axonal transport of alphaherpesviruses provides one possible new antiviral target built on the back of studies on the role of the cytoskeleton in viral replication. Not only would this potentially block establishment of latency in neurons, for which there are currently no drugs but also provide the basis for an attenuated non-neuroinvasive vaccine candidate [157].

This review highlights the known key alphaherpesviral interactions that allow these viruses to manipulate host cells during infection and clearly these pathogens have developed mechanisms to use host cytoskeletal structures at almost all stages of the lifecycle. This includes but is not limited to, actin remodelling during viral surfing prior to entry (and most likely exit), transport along microtubules to deliver viral components to and from the nucleus and for axonal transport during initial viral uptake into the sites of latency or reactivation. Certainly, in the context of cytoskeleton regulation and subversion of molecular motors by alphaherpesviruses our increasing knowledge will not only provide targets for new antivirals but inform on attenuated virus design for gene therapy [191], oncolytic virotherapy [27] and vaccine development [157].

## Figures and Tables

**Table 1:** Alphaherpesvirus-host interactions that regulate actin dynamics.

Virus	Viral Protein	Host Cell Proteins/System	Role	Reference
HSV-1 HSV-2	gD	Nectins	gD binds to host-cell receptor nectin. Nectins regulate actin reorganisation by activating remodelling proteins like Ras/Rho GTPases (Rap1, Cdc42, Rac1). Rac1/Cdc42 have been implicated in signalling during early HSV-1 infection. However, evidence has shown that Rac1 and Cdc42 signalling does not occur in infected keratinocytes [75].	[76,77,78]
HSV-1	gD	Likely nectin-1/HVEM	Following viral binding, there is activation of Cdc42 and RhoA, causing filopodium-like protrusions in corneal fibroblasts and nectin-1-expressing Chinese hamster ovary (CHO) cells. Virus associates with these protrusions during viral entry and actin depolymerisation drugs inhibit viral entry. Also observed in a zebrafish model.	[78,79]
HSV-1	Unknown	Na+/H+ exchangers (NHE), p21-activated kinases	Internalisation of HSV-1 relies on the activity of these NHEs on the plasma membrane of Vero, HeLa, HEp-2 and PtK_2_ cells. These are known to be involved in macropinocytosis, an actin-dependent endocytic process which takes up extracellular fluid and macromolecules. This process can withstand the endocytosis of large structures (0.2–5 μm) which a large pathogen like HSV-1 can exploit.	[80]
HSV-2 PrV	pUS3	PAK1/PAK2	pUS3 directly phosphorylates group A p21-activated kinases (PAKs). Actin stress fibre disassembly during PrV infection of mouse embryonic fibroblast (MEF) and swine testicle (ST) cells is pUS3-mediated and requires PAK2. Cellular projections are mediated by PAK1. pUS3 kinase activity leads to protein kinase A-dependent phosphorylation of RhoA in ST cells; this subverts the antagonistic RhoA and Cdc42/Rac1/PAK signalling cascades for actin remodelling.	[81,82,83,84,85,86]

**Table 2:** Alphaherpesvirus-host interactions that exploit actin-based myosin.

Virus	Viral Protein	Host-Cell Proteins/System	Role	Reference
HSV-1	Unknown	Myosin Va	Myosin Va is activated during infection, facilitating transport of virion- and glycoprotein-bearing vesicles from TGN to plasma membrane through cortical actin in HeLa cells. It is hypothesised that egressing virions (collected within TGN-derived vesicles) behave in a similar manner to other myosin-dependent cargo: kinesin motors (see Section 4) deliver vesicles to cortical actin and myosin Va “captures” the vesicles and then transports them to the plasma membrane.	[98]
HSV-1	pUL49 (VP22)	Non-muscle myosin heavy chain IIA (NM-IIA)	Affinity chromatography experiments with HSV-1-infected baby hamster kidney (BHK) cell extracts have shown tegument protein VP22 interacts with NM-IIA. HSV-1 infection of Vero cells redistributes NM-IIA but only a subpopulation of NM-IIA colocalises with VP22 in a perinuclear cluster.	[99]
HSV-1	gB	NM-IIA/myosin light chain kinase (MLCK)	NM-IIA is a functional coreceptor for gB in Vero cells. Inhibition of NM-IIA (by blebbistatin) and MLCK (by ML-7 and ML-9) decreased viral entry into corneal epithelial cells. Activation of NM-IIA by MLCK is necessary for the cytoskeletal rearrangements needed for HSV-1 infection of corneal cells. To regulate actin, NM-IIA cross-links and contracts F-actin.	[100,101]
HSV-1	gB	Non-muscle myosin heavy chain IIB (NM-IIB)	Interaction may serve as an entry coreceptor in the CV-1 in origin with SV40 genes (COS) cell line as above. Activation of NM-IIB by MLCK is also necessary for the cytoskeletal rearrangements. Likely to be important in a range of cell types.	[102]

**Table 3:** Alphaherpesvirus-host interactions that regulate microtubule stabilisation.

Virus	Viral Protein	Host-Cell Proteins/System	Role	Reference
HSV-1	Capsid (unknown)	Microtubule plus–end tracking protein (+TIP) complex EB1, CLIP-170 and dynactin-1	Studies in normal human dermal fibroblasts (NHDFs) show EB1 directs viral capsid interaction with plus end of microtubules. Stabilises microtubules and recruits molecular motor dynein for retrograde transport during initial viral entry.	[121]
HSV-1	pUS3	Glycogen synthase kinase 3β	pUS3 phosphorylation inactivates the host cell kinase in NHDFs, leading to microtubule stabilisation by +TIP and cytoplasmic linker-associated proteins (CLASPs), to enhance viral spread.	[122]
HSV-1	ICP0	Unknown	ICP0 is a viral E3 ligase which was found to destabilise and unbundle microtubules in Vero cells to aid in viral assembly and egress.	[123]
HSV-1	pUL37	IKAP (Iκβ kinase complex associated protein)	Yeast two-hybrid screening indicated an interaction between tegument protein pUL37 and IKAP. IKAP has proposed roles in microtubule stabilisation [124]. pUL37 binding of the C-terminal region of IKAP could regulate its activity and stabilise cytoskeletal rearrangements during the changes that occur from infection to enhance viral replication. Yet to be tested in cell lines.	[125]

**Table 4:** Alphaherpesvirus-host interactions that exploit microtubule-based motors dynein and kinesin.

Virus	Viral Protein	Host-Cell Proteins/System	Role	Reference
HSV-1	pUL34	Intermediate chain of the dynein complex (IC-1a)	Pulldown experiments of infected Vero and HEp-2 cells with IC-1a (and reciprocal experiments) identified an interaction with pUL34. pUL34 localised to the nuclear membrane when expressed by a baculovirus vector, confirming the protein is involved in transport to the nuclear membrane in the viral context. pUL34 is not a structural protein [64] so unlikely to be involved in cytoplasmic viral capsid transport.	[140]
HSV-1	pUS11	Kinesin-1 (KIF5)	Residues 867–894 of ubiquitous human kinesin-1 bind to a C-terminal RNA-binding domain of tegument pUS11 as evidenced by pulldown assays. HSV-1 pUS11 has 63% homology to HSV-2 pUS11, with variation in the N-terminal half, so this interaction could prove to be transferable. Not confirmed in vivo and one study suggests pUS11 is not a structural tegument protein [64].	[141]
HSV-1	Tegument proteins	Dynein, dynactin, kinesin-1	Tegumented capsids (lacking outer tegument and envelopes) were capable of binding microtubule associated proteins (MAPs) sourced from pig brain cytosol. 10% of capsids tested by in vitro single particle analysis had bound dynein and kinesin-1 simultaneously, suggesting HSV-1 capsid transport is not directed by exclusive presence of either minus- or plus-ended motors. Inner tegument, pUL36 and pUL37, suggested as most likely to bind motors or recruit other tegument proteins that bind motors at this stage, especially with early findings that without pUL36, HSV-1 particles form but have reduced infectivity and a decreased ability to bind to and transport along microtubules [142].	[143]
PrV	pUL36	Dynein, dynactin	Immunoprecipitation of pUL36-transfected HEK293 cells showed that it interacts with dynein/dynactin and can drive transport in the absence of other viral proteins when transfected into Vero cells. pUL36 is capable of transporting viral capsid along microtubules in conjunction with capsid-binding pUL25. A large proline-rich domain in the pUL36 C-terminus contributes to the interaction.	[144]
HSV-1	pUL37	Dystonin/BPAG1	Tegument protein pUL37 recruits dystonin/BPAG1 in human foetal foreskin fibroblasts (HFFF2), which most likely functions to crosslink and stabilise microtubules, to facilitate viral capsid transport during viral entry. Plus-end directed transport is inhibited by dystonin depletion, providing evidence that pUL37-dystonin interaction is required for transport of capsids from the centrosome to the nucleus.	[145,146]
HSV-1	pUS9	Kinesin-1	Five arginine residues in the basic domain of envelope protein pUS9 bind host motor kinesin-1 as determined by truncation construct pulldown studies. This domain was shown to contribute to anterograde axonal transport in infected primary rat dorsal root ganglionic (DRG) neurons and a mouse zosteriform model.	[147]
PrV	pUS9	Kinesin-3 (KIF1A)	pUS9 was found to interact with kinesin-3 using GFP-Trap pulldown. This interaction was shown to mediate efficient axonal sorting and anterograde axonal transport of viral particles in primary rat superior cervical ganglion neurons [148].	[149]
HSV-1	pUL35 (VP26)	Dynein light chains Tctex1 and RP3	In vitro yeast two-hybrid evidence that capsid protein VP26 recruits these dynein light chains. Microinjection of HEp-2 cells with HSV-1 ± VP26 suggested VP26 was important for viral retrograde transport. Subsequent deletion studies in cell lines suggest this is a dispensable interaction [150,151].	[152]
HSV-2	pUL56	Kinesin-3 (KIF1A)	In vitro evidence that envelope protein pUL56 interacts with kinesin-3 with a C-terminal transmembrane domain important for this interaction in transfected Vero cells. Possible role in anterograde axonal transport. Shown in PrV to support virus dissemination in vivo in embryonic chick DRG and an infected mouse model but is dispensable for intra-axonal transport beyond the sorting barrier [153].	[154]
